# No Difference between Percutaneous and Arthroscopic Techniques in Identifying the Calcaneal Insertion during Ankle Lateral Ligament Reconstruction: A Cadaveric Study

**DOI:** 10.1155/2019/2128960

**Published:** 2019-01-29

**Authors:** Hong-Yun Li, Sheng-Kun Li, Ri Zhou, Shi-Yi Chen, Ying-Hui Hua

**Affiliations:** ^1^Sports Medicine Center of Fudan University, Department of Sports Medicine and Arthroscopy Surgery, Huashan Hospital, 200040, Shanghai, China; ^2^Department of Sports Medicine, Peking University Shenzhen Hospital, 518036, Shenzhen, Guangdong, China

## Abstract

*Background. *Both percutaneous and arthroscopic techniques have been introduced in anatomic ankle lateral ligaments reconstruction. The purpose of this study was to compare these two techniques in identifying the calcaneal insertion of the calcaneofibular ligament (CFL).* Methods.* Fifteen fresh-frozen human ankle cadaver specimens were used in this study. Each specimen was tested in three stages. For stage 1, each specimen was evaluated under arthroscopy. After debridement was performed, the insertion of the CFL on the calcaneus was identified, and a 1.5mm Kirschner wire was drilled at the center of the insertion. For stage 2, a percutaneous technique was used to identify the center of the insertion of the CFL. A second 1.5 mm Kirschner wire was drilled through the skin marker. For stage 3, the ankle was dissected, the footprint of the CFL was identified under direct vision, and the distances between the center of the CFL insertion on the calcaneus and the two Kirschner wires were measured, respectively.* Results. *In the arthroscopic technique group, the mean distance from the Kirschner wire to the center of the CFL insertion in the calcaneus was 3.4 ± 1.3 mm. In the percutaneous technique group, the mean distance from the Kirschner wire to the center of the CFL insertion was 3.2 ± 1.4 mm. No significant difference was found between the two groups.* Conclusion. *No difference in identifying the calcaneal insertion of the CFL was found between the percutaneous and the arthroscopic ankle lateral ligaments reconstruction technique. Both techniques can be used during anatomic ligaments reconstruction in treatment of chronic ankle instability.

## 1. Introduction

Lateral ankle sprain is the most common sports-related injury. Thirty-four percent of patients will incur a chronic ankle instability within 3 years after the first ankle sprain [1]. Moreover, 20% to 40% of patients with chronic instability require surgical treatment [2]. Numerous surgical techniques have been introduced to treat chronic lateral ankle instability, including nonanatomical reconstruction, anatomical repair, and anatomical reconstruction. Although nonanatomical reconstruction techniques have resulted in a high degree of patient satisfaction, these reconstructions are prone to numerous complications after surgery. On the other hand, anatomical repair has been widely used with few complications, but it is not suitable for patients with anterior talofibular ligament (ATFL) and/or calcaneofibular ligament (CFL) deficiencies [3]. More recently, anatomical reconstruction has been introduced to anatomically recreate the stability of the ankle joint. Excellent results have been reported following anatomical open reconstruction [3, 4].

In the past decade, ankle arthroscopy has been more and more frequently used in sports medicine. Thereafter, arthroscopic anatomic lateral ankle ligaments reconstruction procedures have been introduced [5–10]. The authors believed that arthroscopic anatomic reconstruction technique is reproducible and safe. The advantages of arthroscopic technique are minimally invasive, preventing injury of blood vessels and nerves, facilitating vascularization and low complications after the operation, and better conserving the proprioceptive properties and shortening operation time [5, 10, 11]. Moreover, previous studies found that more than 90% patients with chronic ankle instability combination intra-articular lesions and the presence of any combination of associated intra-articular lesions might result in a poor outcome [4, 12]. Therefore, the surgeons can document and treat these concomitant intra-articular pathologies during the arthroscopic procedure to improve the clinical functional outcomes.

The shortage of arthroscopic procedure in drilling the calcaneal tunnel is the calcaneal insertion of the CFL is difficult to visualize [13]. Thereafter, previous studies introduced a new, simple percutaneous technique to drill the calcaneal tunnel [14, 15]. They demonstrated that the percutaneous technique was more accurate to identify the location of the calcaneal tunnel than arthroscopic technique. However, to date, no study has been published to compare these two techniques. The purpose of this cadaveric study was to compare this percutaneous technique with arthroscopic reconstruction technique in identifying the calcaneal insertion. It was hypothesized that there is no difference in identifying the calcaneal insertion of the CFL between the percutaneous and the arthroscopic techniques.

## 2. Materials and Methods

### 2.1. Specimens Preparation

Fifteen fresh-frozen human ankle cadaver specimens were obtained and approved for use by the Body Donation Center of the authors' University. The mean age of the patients at death was 62.2years (range: 49–69 years), with nine males and six female's cadaveric ankles obtained. Eight of the ankles were right and seven were left. The ankles were examined visually and radiographically to rule out major ankle pathology. The specimens were fresh frozen and stored at −20°C. Prior to the study, the cadaveric ankles were thawed at 5°C for 24 h. The moisture of the specimens was maintained with saline spray during the preparation and studying phases.

Each specimen was tested in three stages. For stage 1, each specimen was evaluated under arthroscopy. After debridement was performed, the insertion of the CFL on the calcaneus was identified and a 1.5mm Kirschner wire was drilled at the center of the insertion. For stage 2, a percutaneous technique was used to identify the center of the CFL insertion on the calcaneus. A second 1.5 mm Kirschner wire was drilled through the skin marker. For stage 3, the ankle was dissected, the footprint of the CFL was identified under direct vision, and the distances between the center of the CFL insertion and the two Kirschner wires were measured, respectively.

### 2.2. Surgical Techniques

#### 2.2.1. Arthroscopic Technique

The ankle was fixed on a standard extremity holder in supine position without distraction. Standard arthroscopy equipment including 4.0mm, 30° arthroscopy, and 4.5mm shaver was used. Three portals including anterior medial, anterior lateral, and sinus tarsi portals were established, which was described in detail in previous studies [8, 13]. Debridement was performed along the lateral gutter and lateral subtalar joint. The sheaths of peroneal tendons were open to expose the calcaneal insertion of the CFL between the lateral border of the calcaneus and the peroneal tendons [13]. After the footprint of the CFL was identified, a 1.5mm Kirschner wire was drilled at the center of the footprint through the sinus tarsi portal.

#### 2.2.2. Percutaneous Technique

This technique was described specifically in previous studies [14, 15]. The contour of the lateral malleolus was identified. Two lines were drawn with a marker pen. The first line was going down along the posterior cortex of the fibular shaft, and the second line was perpendicular to the first line and running through the tip of the lateral malleolus. The point located 1 cm inferior and 1 cm posterior to the intersection of these two lines indicated the center of the CFL insertion at the calcaneus. A second 1.5 mm Kirschner wire was drilled through this skin marker.

**Figure 1:**
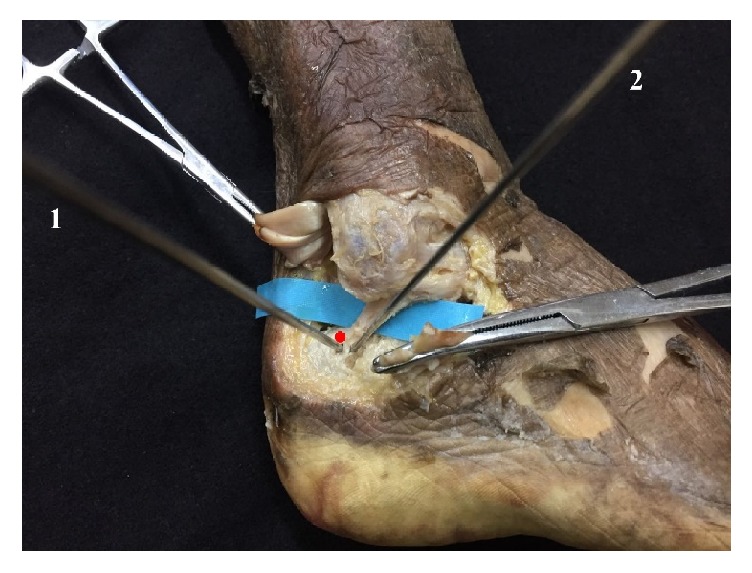
The dissection was performed to expose the CFL insertion at the calcaneus. The distances from these two Kirschner wires to the center of the distal insertion of the CFL were measured. (1) The Kirschner wire drilled through percutaneous technique; (2) the Kirschner wire drilled through arthroscopic technique; red dot: the center of CFL insertion on the calcaneus.

The dissection was then performed to expose the CFL insertion at the calcaneus (Figure 1). The distances from these two Kirschner wires to the center of the distal insertion of the CFL were measured with an electronic digital caliper (accuracy value 0.01 mm) (Shanghai Tool Works Co., Ltd. Shanghai, China).

A senior surgeon specializing in ankle arthroscopy and sports medicine performed all the operations to avoid the bias.

Each measurement was repeated 3 times for intraobserver analysis as well as for calculation of the mean values. As a test of interobserver, two of the authors (the first and the second authors) measured the specimens independently.

### 2.3. Statistical Analysis

A priori power analysis was used to calculate the sample sizes. According to previous study [15], the minimum difference was selected to be 2.4 mm and a standard deviation of 1.8 mm was assumed in each group. For a power of 0.8 and a significance level of 0.05, 11 specimens in each group were needed. The paired t test was used. Intra- and interobserver reliabilities for measurement were analyzed using Intraclass Correlation Coefficient (ICC). An ICC of <0.4 was considered poor, between 0.4 and 0.7 moderate, and >0.7 excellent. A reliability analysis of scale was used to calculate the ICC values. Data were presented as the mean ± standard deviation (SD). Differences were considered to be statistically significant for P values of < 0.05. All statistical analyses were conducted using SPSS 19.0 (IBM Corporation, Armonk, New York, USA).

## 3. Results

The intra- and interobserver reliabilities were 0.987 and 0.942, respectively. In the arthroscopic technique group, the mean distance from the Kirschner wire to the center of the CFL insertion in the calcaneus was 3.4 ± 1.3 mm. The distances varied from 1.3 mm to 5.8 mm. In the percutaneous technique group, the mean distance from the Kirschner wire to the center of t the CFL insertion was 3.2 ± 1.4 mm. The distances varied from 1.0 mm to 4.1 mm. No significant difference was found between two groups. Moreover, in the arthroscopic group, 9 of 15 Kirschner wires were anterior and 14 of 15 Kirschner wires were inferior to the center of the CFL footprint. In the percutaneous technique, 12 of 15 Kirschner wires were posterior and 13 of 15 Kirschner wires were inferior to the center of the CFL footprint.

## 4. Discussion

The most important finding of the present study was that there was no difference in identifying the calcaneal insertion of the CFL between the percutaneous and arthroscopic techniques. As anatomic lateral ligaments reconstruction to chronic ankle instability provides satisfactory clinical outcomes, more and more studies introduced the arthroscopic techniques to reconstruct ankle lateral ligaments [5–10, 14]. The goal of the arthroscopic technique was to reduce the morbidity of the surgical procedure without change the original anatomic attachments. Previous studies indicated this technique was reproducible and safe. Moreover, it allows the surgeons to evaluate the associated injuries with less postoperative morbidity [13, 16]. One of the shortages of arthroscopic reconstruction technique is the calcaneal insertion of the CFL is difficult to visualize. Only after debriding the lateral side of the sinus tarsi and opening the sheaths of the peroneal tendons could the insertion of CFL in the calcaneus be identified. Another shortage is the accuracy of arthroscopic identifying the center of CFL footprint is not high. In a cadaver study, the calcaneal tunnel drilled under arthroscopic monitoring was 3.3 ± 2.8 mm too anterior from the CFL calcaneal footprint [13]. Thereafter, more recently studies introduced a new percutaneous technique to make the calcaneal tunnel [14, 15]. The mean distance from the Kirschner wire drilled through percutaneous technique to the center of the distal insertion of the CFL was 2.4 ± 1.8 mm. The authors believed this technique was more accurate to identify the location of the calcaneal tunnel. Moreover, it was a very simple, reproducible, and safe technique [15]. Other advantages of this percutaneous technique included preserving tissue structure, unnecessary adjustment of the body position, and no additional transplant device requirement [14]. In the current study, we compared percutaneous and arthroscopic technique in identifying the footprint of the CFL at the calcaneus. We found there was no significant difference in identifying the calcaneal insertion of the CFL between the two techniques.

Previous studies indicated that CFL is very important for lateral ankle stability [17–19]. It plays a major lateral stable role when the ankle is in the neutral and dorsal flexion position [20]. Moreover, the CFL could provide lateral stability of the subtalar joint [21]. Previous studies indicated that subtalar instability might occur if the CFL was disrupted [21, 22]. Therefore, it is very important to restore the normal anatomy as much as possible for better functional outcome and normal ankle mechanic, to prevent ankle reinjury and early development of osteoarthritis.

Many previous studies described the anatomic reference to identify the CFL footprint during anatomical CFL reconstruction. Neuschwander et al. [23] found the CFL footprint on the calcaneus was almost 3 cm posterior and superior to the peroneal tubercle. Best et al. [24] concluded that the distal insertion of the CFL could be identified on lateral X-rays at the intersection of two lines. One line was a vertical tangent line from the posterior convexity of the talus, and the second line was a perpendicular tangent line from the deepest visible concavity of the tarsal sinus. Glazebrook et al. [25] described that the center of the CFL footprint was at 13 mm distal and on the vertical line down from the center of the subtalar joint. However, for a surgeon, it is difficult to identify the center of the CFL insertion during the surgical procedure using these methods. Therefore, we believe the percutaneous technique used in the current study was an easy, reliable, and reproducible technique to make the calcaneal tunnel.

One of the shortages of percutaneous technique was that variable insertion of the CFL has been found. The variable insertions result in variable obliquity and angles of the ligament relative to the long axis of the fibula, coronal, and sagittal planes [26, 27]. Clanton et al. [28] found that the CFL originated an average of 5.3 mm (95% CI, 4.2 to 6.5) from the inferior tip of the lateral malleolus at the anterior fibular border and inserted an average of 16.3 mm (95% CI, 14.5 to 18.1) from the posterior point of the peroneal tubercle. These variables limit the use of percutaneous technique. Secondly, the risk of injury sural nerve is relatively high when using percutaneous technique [15, 29]. Therefore, careful “nick and spread" technique should be used to avoid injury the nerve.

In the current study, most Kirschner wires in the arthroscopic technique group were anterior and inferior to the center of the CFL footprint, and most Kirschner wires in the percutaneous technique were posterior and inferior to the center of the CFL footprint. Therefore, the results can help us to improve the accuracy in making calcaneal tunnel.

The main limitation of the current study was that we did not measure the characteristics of the CFL, the footprint of CFL in relation to bony landmarks, and the distance between the insertion and the surrounding nerves and blood vessels. However, these characteristics were measured repeatedly in many previous studies; it is not necessary to repeat the same work. On the other hand, after arthroscopic procedure, the irrigation fluid penetrated the surrounding tissue, which made it inaccurate to measure the distance between the Kirschner wire and the surrounding nerves and blood vessels. In the future work, paired ankles may be needed to compare these two techniques.

## 5. Conclusion

In this cadaveric study, no difference in identifying the calcaneal insertion of the CFL was found between the percutaneous and the arthroscopic ankle lateral ligaments reconstruction technique. Both techniques can be used during anatomic ligaments reconstruction in treatment of chronic ankle instability.

## Data Availability

The data used to support the findings of this study are available from the corresponding author upon request.
